# Effects of Fine Particulate Matter on* Pseudomonas aeruginosa* Adhesion and Biofilm Formation* In Vitro*

**DOI:** 10.1155/2018/6287932

**Published:** 2018-07-04

**Authors:** Seon Hee Woo, Sang Moog Lee, Ki Cheol Park, Gyeong Nam Park, Byeolnimhee Cho, Insoo Kim, Jinwoo Kim, Sungyoup Hong

**Affiliations:** ^1^Department of Emergency Medicine, Incheon St Mary's Hospital, The Catholic University of Korea College of Medicine, Seoul 06591, Republic of Korea; ^2^Department of Anesthesiology and Pain Medicine, Daejeon St Mary's Hospital, The Catholic University of Korea College of Medicine, Seoul 06591, Republic of Korea; ^3^Clinical Research Institute, Daejeon St Mary's Hospital, Daejeon 34943, Republic of Korea; ^4^Department of Emergency Medicine, Daejeon St Mary's Hospital, The Catholic University of Korea College of Medicine, Seoul 06591, Republic of Korea; ^5^Department of Emergency Medical Service, Daejeon Health Institute of Technology, Daejeon 34504, Republic of Korea

## Abstract

Respiratory infections of* Pseudomonas aeruginosa* are a major cause of mortality and morbidity for hospitalized patients. Fine particulate matter (FPM) is known to have interactions with some bacterial infection in the respiratory system. In this report, we investigate the effect of different concentration of FPM on* P. aeruginosa* attachment and biofilm formation using* in vitro* cell culture systems.* P. aeruginosa* were cultured to form mature biofilms on hydroxyapatite-coated peg and the number of bacteria in the biofilms was enumerated. Morphology of biofilm was imaged with scanning electron microscopy and confocal laser scanning microscopy. Bacterial affinity change to the cell membrane was evaluated with attached colony counting and fluorescence microscopy images. Alteration of bacterial surface hydrophobicity and S100A4 protein concentration were explored as mechanisms of* P. aeruginosa* adhesion to human cells. There were a concentration-dependent increase of thickness and surface roughness of biofilm mass.* P. aeruginosa* adherence to respiratory epithelial cells was increased after FPM treatment. Bacterial surface hydrophobicity and S1000A4 protein concentration were increased with proportionally the dose of FPM in media. FPM in the airway could enhance both the adhesion of* P. aeruginosa* to epithelial cells and biofilm formation. Bacterial surface hydrophobicity and human cell plasma membrane injury are associated with binding of* P. aeruginosa* on airway epithelial cells and biofilm formation.

## 1. Introduction

The facultatively anaerobic Gram-negative bacilli* Pseudomonas aeruginosa* is the most common causative agent of ventilator-associated pneumonia, showing a global prevalence rate of 26% [1]. Pulmonary infection by* P. aeruginosa* is characterized by progressive obstructive disease through the development of biofilms, particularly in patients with cystic fibrosis (CF) and immunocompromised patients [2, 3]. Biofilm formation is a complex phenomenon involving the initial attachment of planktonic bacteria and production of an extracellular polysaccharide matrix [4]. A surface can become covered by a film of microbial cells and macromolecules including polysaccharides, proteins, nucleic acids, uronic acids, and humic-like substances [5]. Consequently, the physicochemical properties of the surfaces are modified, potentially affecting the settlement of various microfoulers, whose populations may increase markedly within a few days [6].

Fine particulate matter (FPM) with an aerodynamic diameter of ≤2.5 *μ*m (PM2.5) is known to be associated with a variety of adverse health effects [7–10]. A nationwide study in the USA, which involved an open cohort of all Medicare beneficiaries, reported that adverse effects of PM2.5 exposure with increases of 10 *μ*g/m^3^ were associated with an increase of 7.3% in the all-cause mortality rate [11]. A concentration increase of 10 *μ*g/m^3^ for PM2.5 was associated with an increase of 1.05% (95% confidence interval [CI]: 0.95%, 1.15%) in the daily mortality rate [12].

From the perspective of respiratory disease, FPM extensively penetrates the respiratory system and exacerbates lung disease in affected individuals [10, 13, 14]. Exposure to FPM is associated with accelerated bacterial colonization and aggravated respiratory infection, including pneumonia [15–17]. Psoter et al. [18] reported that each 10 *µ*g/m^3^ increase in FPM exposure is associated with an increased risk of* Pseudomonas* infection, with a hazard ratio of 1.24 in young children with CF. Li et al. [19] reported that FPM in the airways of mice showed exacerbated lung injury and altered the T-cell balance.

Only one study has reported that particulate air pollutants cause structural changes in the biofilms of Gram-positive* Staphylococcus aureus* [20]. However,* S. aureus* is involved in only low proportion of CAP incidence [21]. Thus, we investigated the effects of FPM on contour and surface morphological changes of biofilm by* P. aeruginosa* by electron microscopy (EM) and confocal laser microscopy (CLSM) to quantitatively measure biofilm thickness.

The mechanisms underlying aggravation of bacterial infection by FPM exposure remain unclear [20]. Therefore, we predicted that FPM exposure to respiratory epithelium stimulates bacterial adhesion and infection in human cells. To clarify this hypothesis, the adhesion and infectivity of* P. aeruginosa* were assessed by labeling bacteria with the fluorescent dye SYTO 9 [22].

High bacterial wall hydrophobicity is associated with enhanced adhesion to mineral particles [23]. The authors also speculated that changes in membrane hydrophobicity from FPM impact bacterial biofilm formation. Subsequently, a change in the bacterial surface charge due to FPM may promote binding of bacteria to human cells. Experiments involving the adherence of bacteria to hydrocarbons with simple measurability for overall measurement of bacterial cell surface hydrophobicity were performed [24].

S100A4, a calcium-binding protein, has been implicated in cell membrane injury. A previous study reported that S100A4 protein was overexpressed in airway epithelial cells after lung injury in animal experiments [25]. Another study using live cell imaging of plasma membrane repair showed that S100A4 accumulated at the lung injury site [26]. We also examined the S100A4 protein concentration in the cell culture supernatant as a marker of putative cell membrane destruction in a model of airway epithelial cell injury due to FPM.

## 2. Materials and Methods

### 2.1. Bacterial Strains, Cell Line, and Chemicals

All materials were purchased from Sigma-Aldrich (St Louis, MA) unless specified otherwise. Six* P. aeruginosa* colonies acquired from pneumonia patients in Korea were purchased from the Korean National Research Resource Center (Seoul, Korea). All bacteria were stored in tryptic soy broth (TSB, BD Diagnostics, Franklin Lakes, NJ) with 15% glycerol at -80°C and were thawed before culture. Standard reference material 2786 (SRM 2786) with mean particle diameter < 4 *µ*m was purchased from the National Institute of Standards and Technology (NIST; Gaithersburg, MA). The human alveolar epithelial cell line A549 cell line was purchased from the Korean Cell Line Bank (Seoul, Korea). RPMI1640 with L-glutamine (300 mg/L), 25 mM HEPES, and 25 mM NaHCO3 was purchased from the American type culture collection (ATCC; Manassas, VA).

### 2.2. Biofilm Formation of P.* aeruginosa*

A 0.5 Mcfarland standard suspension of* P. aeruginosa* was added cell culture treated plates with flat-bottom wells (Corning Costar 3596; Corning, NY) containing serially diluted FPM (0-100 *μ*g/mL) in Mueller-Hinton broth (MHB, BD Diagnostics, Franklin Lakes, NJ). Furthermore, the MBEC™ Biofilm Inoculator (Innovotech, Edmonton, Canada) pegs coated with hydroxyapatite, facilitating bacterial biofilm growth, were immersed into the bacterial suspension. Biofilms were established on the pegs under batch conditions (no flow of nutrients) for 18 h at 37°C with gentle agitation at 120 rpm.

### 2.3. Isolation and Enumeration of Bacteria in the Biofilm

Enumeration of the* P. aeruginosa* cells in the biofilms was following previous study [27]. The cultured pegs and plates were rinsed with phosphate-buffered saline (PBS) and placed in a 0.1% (w/v) crystal violet (CV, Duksan pure chemicals Co. Ltd., Ansan, Korea) solution for 15 min, rinsed again, and dried for several hours in a biological safety cabinet (BSC). The pegs and plates with CV-stained biofilms were immersed in 95% ethanol for 15 min to solubilize the CV. Absorbance of solubilized CV in ethanol was measured at 570 nm using a Sunrise microplate reader (Tecan, Männedorf, Switzerland). The blank control peg and plate samples with only FPM were stained and absorbance was measured. Mean absorbance of control samples was subtracted from the absorbance of each sample with FPM and bacteria for final comparisons.

### 2.4. Scanning Electron Microscopy (SEM)

Pegs with matured biofilm were fixed with a primary fixative (5% glutaraldehyde in 0.1 M Na cacodylate buffer; pH 7.5) at 4°C for 24 h. The peg samples were then progressively dehydrated through 50, 75, 90, 95, and 100% ethanol and dried for 24 h in a BSC. The peg samples were mounted on an aluminium stub and coated with gold using a silver sputter coater (EMS150R S, Electron Microscopy Sciences, Hatfield, PA). Images were acquired using JSM-6300F field emission SEM (Japan electron optics laboratory Ltd., Japan) in wet mode at ~4 Torr and 5°C, using an accelerating voltage of 10 kV at × 5000 magnification [28].

### 2.5. Confocal Laser Scanning Microscopy (CLSM)

Wells on a Perfecta3D® hanging drop plate (Sigma-Aldrich, St. Louis, MO, USA) were inoculated with MHB with bacteria (10^6^ cells/mL) with or without FPM and the plate was inverted and incubated at 37°C for 18 h. Static bacterial biofilm formed on the wells of the drop plate were stained with SYTO9 (5 mM, Sigma-Aldrich) and propidium iodide (PI, 20 mM, Sigma-Aldrich) per 1 mL of PBS at room temperature in the dark for 15 min. After washing with PBS to remove unbound stain three times, the stained biofilms were examined under a Zeiss LSM 710 laser scanning confocal microscope (Oberkochen, Germany).

### 2.6. Bacterial Adhesion and Cellular Invasion

*P. aeruginosa* adhesion to A549 cells was evaluated as reported previously [22]. Monolayers of A549 cells were grown in 96-well plates washed with PBS for four times before the adhesion test. Bacterial suspensions in serum-free RPMI without antibiotics (~2.0 × 10^7^ colony-forming units (CFU)/mL) were added with different concentrations of FPM on the cell monolayer and incubated for 6 h at 37°C. After four washes with PBS, numbers of cell-invading bacteria were determined after cell lysis by exposure to the cell lysis solution. Percentage adhesion was calculated with the following formula:

(1) Number  of  adherent  and  invading  bacteria  CFU/mlNumber  of  bacteria  in  innoculum  CFU/ml×100.

### 2.7. Fluorescence Microscopic Imaging

Bacteria were grown in MHB to mid-exponential phase and were centrifuged at 3000 × g for 5 min, washed twice with PBS, and stained with 0.5 *μ*M SYTO 9 green dye (Thermo Fisher Scientific, Waltham, MA). After three washes with PBS, bacterial pellets were suspended in serum-free RPMI media at approximately 2.0 × 10^7^ CFU/mL. A549 cells were cultured in Lab-TEK six-well chamber slides (Nunc, Naperville, IL) until confluent. Cultures were inoculated with stained bacterial suspension and incubated at 37°C for 6 h. After four PBS washes, the A549 cell monolayer were imaged using a fluorescence microscope (Eclipse TE300, Nikon, Japan) used 488-nm excitation and 520-nm emission filter.

### 2.8. Measurement of Bacterial Surface Hydrophobicity

Bacterial surface hydrophobicity was measured using a modified microbial adhesion to hydrocarbon (MATH) assay using a previously described method [2]. Bacterial cells (OD600 = A1) were suspended in 2.5 mL of PBS. Para-xylene (0.5 mL) was added and the suspensions were incubated at 44°C for 10 min. Samples were homogenized for 60 s and incubated for 1 h to obtain separate organic and aqueous phases. Optical density of the aqueous phase was measured spectrophotometrically at 600 nm (A2). The percentage of bacterial adhesion to hydrocarbons was calculated using the following formula: A (%) = [(A1 − A2)/A1] × 100%.

### 2.9. S100A4 Measurement

A549 cells were cultured with FPM for 4 h and S100A4 levels in the media were determined using a human Sandwich ELISA Kit (LS -F9180, LSBio, Seattle, WA) after centrifugation, in accordance with the manufacturer's instructions. Briefly, samples containing cell culture supernatants were incubated with S100A4 specific capture antibody coated on well. After washing, the wells were incubated with biotin-conjugated anti-S100A4 primary antibodies and followed by washing and incubation with streptavidin-HRP complex. Optical density (OD) of each well was determined at 450 nm using the same Sunrise microplate reader described above.

### 2.10. Statistical Analysis

All experiments were performed in triplicate with duplicated samples (*n* = 6) to evaluate variations in biofilm assay conditions. Differences in biofilm formation, bacterial attachment, and bacterial surface hydrophobicity with differences in FPM concentration were assessed using Kruskal-Wallis test. All statistical procedures were performed using RStudio Version 0.98.932 (Boston, MA). Statistical significance was defined by p values less than 0.05. All data were presented as mean ± standard deviation (SD).

## 3. Results and Discussion

### 3.1. FPM Ameliorated* P. aeruginosa* Biofilm Formation

**Figure 1:**
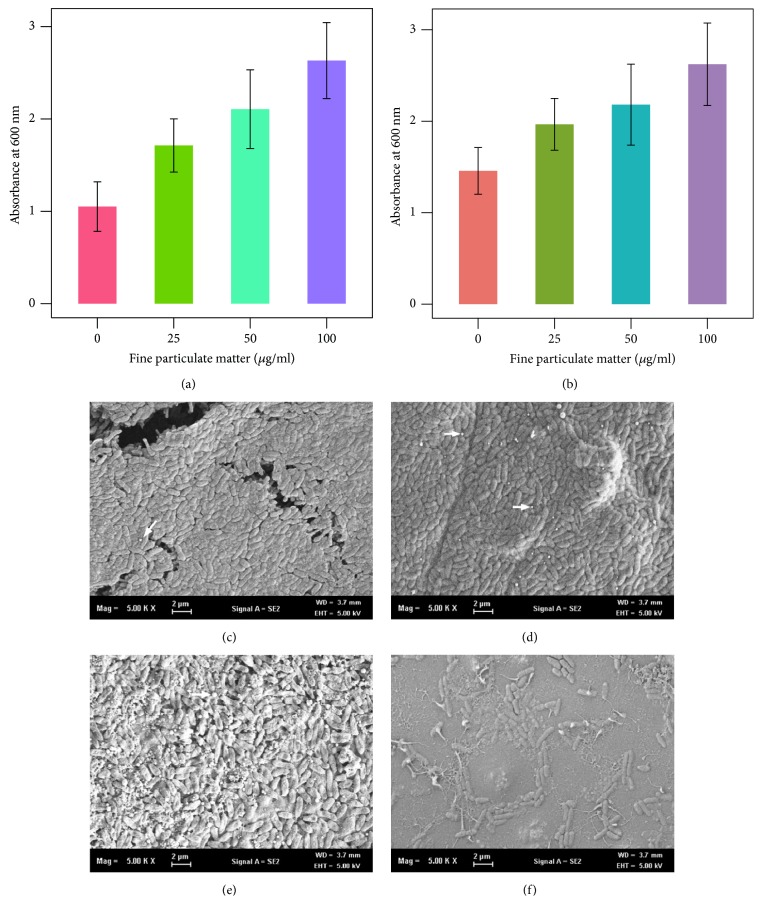
*P. aeruginosa* biofilm formation on peg and flat-bottom well after fine particulate matter (FPM) treatment. (a, b) Amounts of biofilm masses were measured with the absorbance value of crystal violet solubilized from the stained biofilm. Biofilm masses on the peg (a) and flat-bottom well (b) increased with an increase in the concentration of FPM in a dose-dependent manner. Contour and architecture of biofilms were examined using scanning electron microscopy. (c) Dense biofilm and cracked surface are observed in the image after treatment with 100 *μ*g/mL FPM. (d, e) Surface of the peg coated with hydroxyapatite covered with attached bacteria to form a biofilm after treatment with 50 and 25 *μ*g/mL FPM. (f) Bacteria rarely adhere on the peg surfaces and hydroxyapatite-coated surface can be seen in FPM-untreated cultures.

*P. aeruginosa* biofilms formed on the pegs and flat-bottom wells after culture with or without FPM (Figure 1). As the concentration of FPM increased, the density of biofilms also increased on the pegs (p < 0.01, Figure 1(a)) and flat-bottom wells (p = 0.01, Figure 1(b)).* P. aeruginosa* biofilms yielded a dense surface shape on the entire surface of the peg following treatment with 100 and 50 *μ*g/mL FPM (Figures 1(c) and 1(d)). Biofilm formed on the peg was less dense at a concentration of 25 *μ*g/mL FPM (Figure 1(e)). Biofilms formed on the pegs without FPM, however, sparsely covered the hydroxyapatite-coated surface (Figure 1(f)).

**Figure 2:**
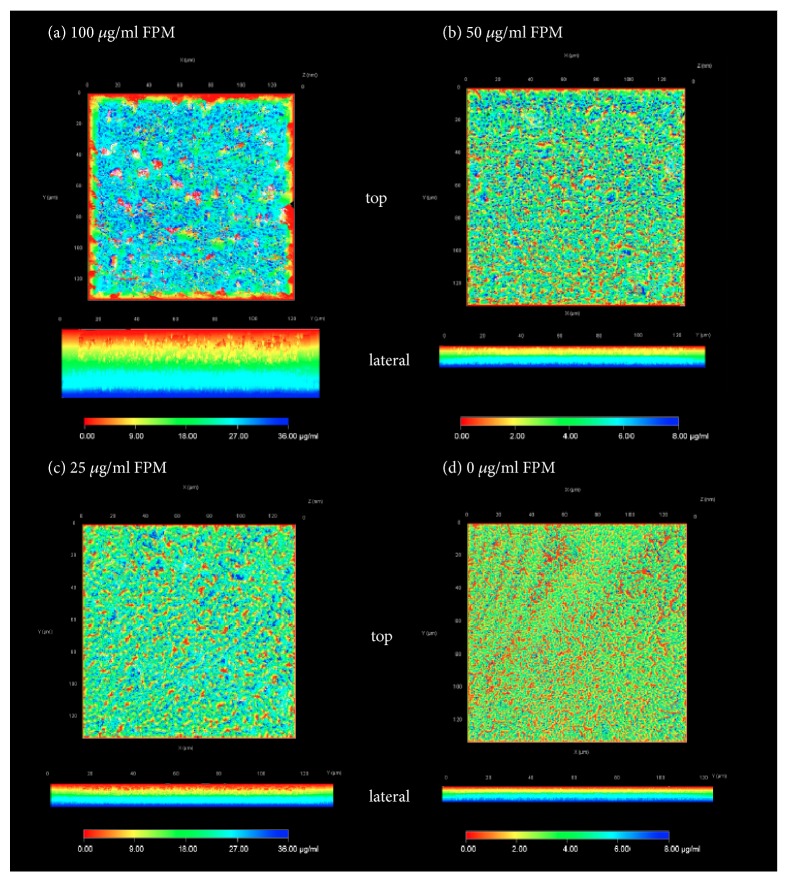
Three-dimensional depth coding images of a* Pseudomonas aeruginosa* biofilm were obtained with CLSM after staining with SYTO9/PI. The upper picture in each image panel shows the top view of the biofilm and the lower picture shows the lateral view of the biofilm depth coding. (a) Figure 2(a) shows the top view and lateral view of the biofilm with a thick texture, coarse surface, and red colored protruding biofilm mass after 100 *μ*g/mL FPM treatment. (b, c) The biofilm became thinner and less prominent and the depth of space in the biofilms was shallow after 50 or 25 *μ*g/mL FPM treatment. (d) The thinnest biofilm and shallow nadirs around the projections were on the well without FPM.

**Figure 3:**
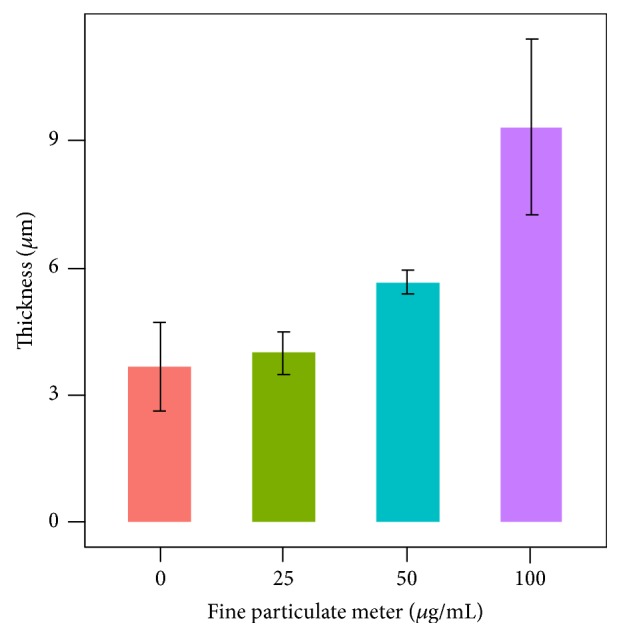
Biofilm thickness was measured with confocal laser scanning microscopy (CLSM). FPM augmented the thickness of* P. aeruginosa *biofilm.

We also measured the thickness of mature biofilms by CLM, which ranged from 2 to 24 *μ*m. Significant thick and rugged surface contours were observed on the biofilm formed on the well bottom in the 100 *μ*g/mL FPM treatment group (Figure 2). The thickness of biofilm masses significantly decreased as the concentration of FPM decreased (p < 0.05, Figure 3).

The present results suggest that the propensity of biofilm formation by* P. aeruginosa* strains increased in the presence of FPM. A recent study reported that black carbon nano-powder significantly amplified bacterial biofilm formation [20]. Black carbon altered the architecture of gram-positive* Streptococcus pneumoniae* biofilms, resulting in a thicker biofilm with irregular protrusions and channels. Those results are consistent with those obtained in the present study, except that we observed dense biofilm with a smooth surface contour on the biofilm by SEM. This difference may be attributed to the bacterial species used and to the floating peg device kept under constant shaking. FPM could be precipitated at the bottom of the plate and may have caused microscopic morphological changes in the biofilms. However, it is difficult for the FPM to attach to the buoyant peg surface during shaking. Our CLSM results agree with the results of a study by Hussey et al. [20] in terms of the increased thickness and surface irregularity of the biofilm assembly.

Goldstein-Daruech et al. [29] reported that clinical isolates from smokers produced significantly larger biofilm and tobacco smoke exposure to smoke-naïve bacteria enhanced biofilm formation in 3 h* in vitro*. Concurrent with the study,* P. aeruginosa* displayed increased propensity for biofilm formation upon FPM treatment in this study. Kleeman et al. [30] reported that particles in cigarette smoke comprise similar organic compounds and have a larger size than particles from burning wood. We used finer particles (0.1–0.2 *µ*m in diameter) than those in cigarette smoke to investigate their effects on bacterial biofilm formation in the small airway, since the finer particles could penetrate finer airways.

Infections by* P. aeruginosa* result in chronic airway inflammation and are associated with morbidity and mortality in CF patients [31] and with ventilator-associated pneumonia [1]. Therefore, the present study aimed to investigate the effects of different concentrations of FPM on* P. aeruginosa* biofilm formation on human cells and associated underlying mechanisms for the same.

### 3.2. FPM Increased* P. aeruginosa* Adhesion on A549 Cells

**Figure 4:**
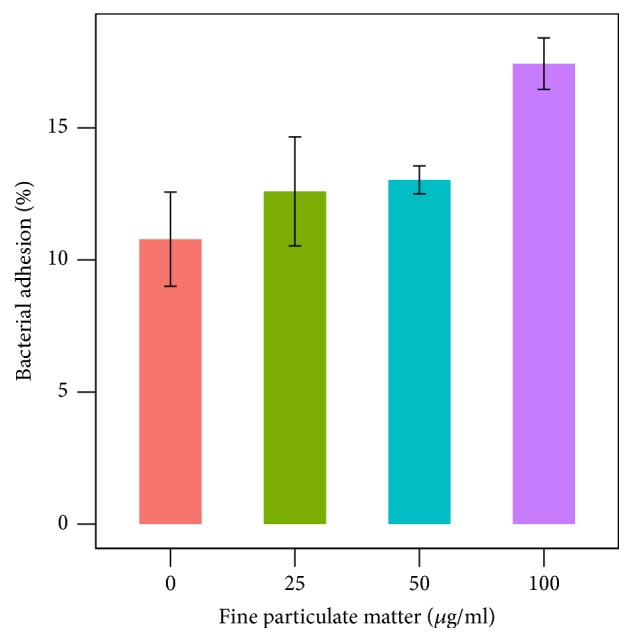
Infection of A549 cells with* P. aeruginosa* stained with SYTO9. Proportions of* P. aeruginosa* adhesion to A549 cells increased after increase of the concentration of fine particulate matter.

We tried to measure* P. aeruginosa* adhesion to human alveolar epithelial cell by plate counting method. Higher concentrations of FPM in the bacterial culture media significantly increased the proportion of adhesive bacteria to human cell surfaces (p<0.01; Figure 4).

Bacterial adhesion, the initial stage of biofilm formation, is nonspecific and reversible. And the damaged epithelia of patients with underlying medical conditions provide an ideal environment for bacteria to form a biofilm. Once transiently adhered to the surface, bacteria synthesize an insoluble extracellular polymeric substance that envelopes the adherent bacteria in a three-dimensional matrix [31].

**Figure 5:**
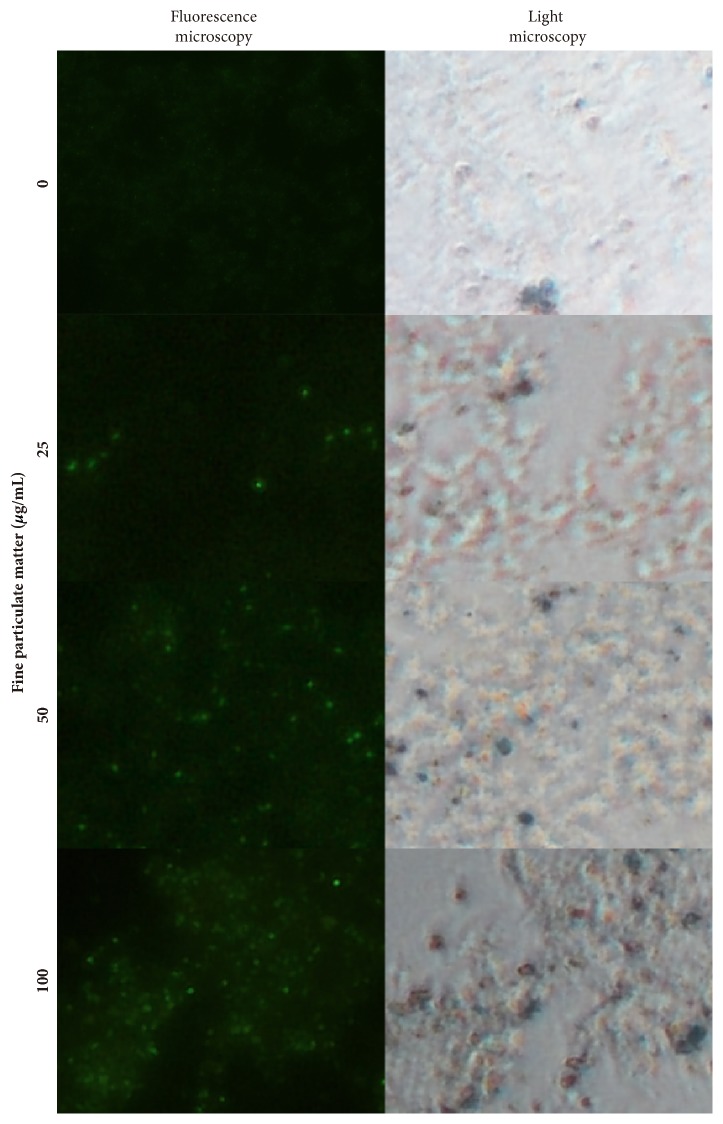
A549 cells infected with* P. aeruginosa* images stained with SYTO9 (green) for 6 h. Images were captured using a fluorescence microscope.* P. aeruginosa* with green fluorescence are rarely observed in a selected microscopic field without fine particulate matter (FPM); however, the number of infecting bacteria increased with an increase in the concentration of FPM in culture media. Original magnification: ×400.

Fluorescent images of A549 cells captured at 6 hours after infection with SYTO9 stained bacteria are shown in Figure 5. Higher concentrations of FPM increased adhesion and infection of* P. aeruginosa* on cells. These phenomena coincide with the findings reported by Mushtaq et al. [32]. They reported that PM10 (particulate matter with an aerodynamic diameter of 10 *μ*m or less) and PM2.5 increased adhesion and infection of* Streptococcus pneumoniae* to A549 airway epithelial cells. To our knowledge, the present study is the first report suggesting that Gram-negative bacterial adhesion is influenced by PM.

### 3.3. FPM Increased Bacterial Surface Hydrophobicity

**Figure 6:**
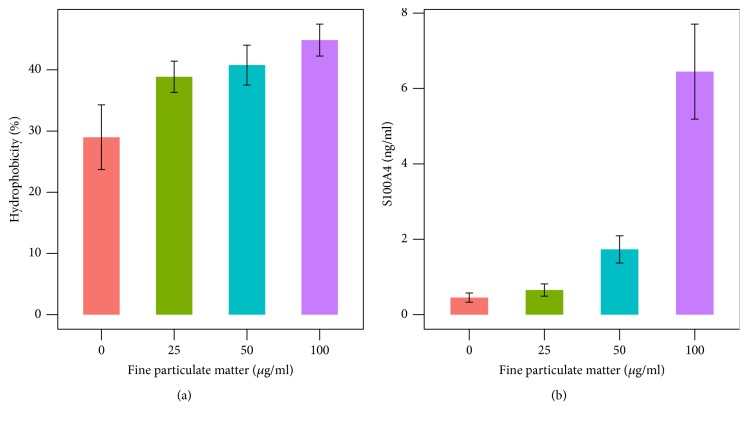
Bacterial surface hydrophobicity and S100A4 protein concentration after treatment of fine particulate matter (FPM). (a)* P. aeruginosa* surface hydrophobicity increased linearly with an increase in FPM concentration. (b) Concentration of S100A4 protein in cell culture supernatant increased with an increase of FPM concentration in a dose-dependent manner.

Although nonspecific attachment of bacteria onto a surface is the initial stage of biofilm formation and is a key determinant in subsequent steps, the mechanism underlying nonspecific bacterial attachment to surfaces is unclear. We showed for the first time that surface hydrophobicity of* P. aeruginosa* clinical isolates increased significantly in FPM treated cultures (p=0.01) in accordance with the mass of the formed biofilm. (Figure 6(a)).

We found that bacterial surface hydrophobicity increased gradually in the presence of FPM in proportion to the mass of the formed biofilm. This finding supports the earlier reports that bacterial adhesion to the surface can be reinforced by changes in bacterial surface hydrophobicity. A previous study reported that higher degree of bacterial surface hydrophobicity is associated with higher bacterial adhesiveness. [24]. Two other studies reported that bacterial surface hydrophobicity is an important factor promoting biofilm formation and binding of antimicrobial agents [33, 34]. Di Bonaventura et al. [35] reported that biofilm formation in Listeria is significantly influenced by bacterial surface hydrophobicity. Bujdáková et al. [36] reported that the gene responsible for cell surface hydrophobicity is upregulated during the formation of a sessile biofilm in comparison to that of the planktonic culture.

These findings are concurrent with those of the present study, wherein anionic charge on the bacterial surface was concealed upon presence of FPM, such that more bacteria adhered to the surface and biofilm formation was enhanced. However, other studies have reported vague data regarding the association between microbial attachment and bacterial surface hydrophobicity [37, 38].

Maricq [39] reported that 60–80% of particles emitted by a diesel engine contain nearly equal number of positive and negative charges. Another study that analyzed emitted particles in Asia reported a noticeable anion deficit and a surplus of cations, especially in urban areas. When bacterial cell was treated with calcite, the zeta potential shifted sharply to the positive direction at all pH conditions. The calcite zeta potential shift may be attributed to the adsorption of the exo-polysaccharides secreted by the cell onto the calcite surface, increasing the negative charges on this surface. This result is similar to previous findings that calcite fine particles neutralizes the negative charge on the bacterial surface and increases bacterial surface hydrophobicity at a steady pH level [40].

### 3.4. FPM Augmented Human Cell Plasma Membrane Damage

The present study also aimed to determine additional factors enhancing the adhesion of* P. aeruginosa*, such as epithelial cell injury resulting from FPM treatment. We found that bacteria attached with human alveolar epithelial cell surfaces more actively when cultured in the presence of FPM.* P. aeruginosa* rarely attached to uninjured airway cells in the normal healthy tracheobronchial tree; however, they could colonize the airways of patients with chronic lung diseases, accompanied with shedding of the epithelium and unmasking receptors for adherence [41] It is known that* P. aeruginosa* has the affinity to bind to the inflamed or injured epithelial cells while the bacteria rarely binds to uninjured intact normal airway epithelial surfaces. [42–44].

Breznan et al. [45] performed the cytotoxic analyses of eight samples of the PM collected from different sources using lung epithelial cells and reported significant elevations in lactate dehydrogenase (LDH) levels in the culture media. Mushtaq et al. [32] reported that PM stimulation did not increase LDH release and disregarded the possibility that increased pneumococcal adhesion is due to cell death or injury. The discrepancy between these two studies may be due to the difference in bacterial species, composition of PM and intracellular characteristics of LDH. LDH is a cytoplasmic enzyme that is present in intracellular space and LDH is only released into the cell culture supernatant when the plasma membrane is disrupted.

Bacteria preferentially have affinity to injured cell membrane and not to intracellular components [41]. Hence, we attempted to determine the levels of S100A4 protein in culture media as marker of damage of A549 cells instead of LDH. S100A4 levels in the supernatant of A549 cell cultures increased significantly in a dose-dependent manner after FPM treatment (p<0.01, Figure 6(b)). S100A4 is a small protein (10–14 kDa) is expressed on the cell surface of A549 cells and upregulated at the site of plasma membrane injury [46], while LDH is leaked from only lysed cells. Together, our findings show linear increases in S100A4 levels after FPM treatment, thereby supporting the hypothesis that FPM causes injury in human cells, thereby enhancing bacterial adhesion to the human cells.

This study has the following limitations. The present study involved exclusively an* in vitro* model; hence, the effects of additional factors in vivo, including adhesion molecules and inflammatory mediators, on bacterial attachment and biofilm formation could not be determined in this study. Higher clinical relevance would be attained from in vivo studies. Furthermore, the electrostatic and chemical properties and the size of FPM in the air may change upon solubilization in culture media; hence, these effects need to be considered as well.

## 4. Conclusions

In conclusion, FPM in the airways could enhance both the binding of* P. aeruginosa* to epithelial cells and biofilm formation. Bacterial surface hydrophobicity and human cell plasma membrane injury enhance bacterial adhesion and biofilm formation.

## Data Availability

All data generated or analyzed during this study are included in this published article.
